# Associations between schizophrenia genetic risk, anxiety disorders and manic/hypomanic episode in a longitudinal population cohort study

**DOI:** 10.1192/bjp.2018.227

**Published:** 2019-02

**Authors:** Alexander Richards, John Horwood, Joseph Boden, Martin Kennedy, Ruth Sellers, Lucy Riglin, Sumit Mistry, Hannah Jones, Daniel J. Smith, Stanley Zammit, Michael Owen, Michael C. O'Donovan, Gordon T. Harold

**Affiliations:** 1 Research Associate, Division of Psychological Medicine and Clinical Neurosciences, Cardiff University, UK; 2 Professor, Christchurch Health and Development Study, Department of Psychological Medicine, University of Otago Christchurch, New Zealand; 3 Associate Professor, Christchurch Health and Development Study, Department of Psychological Medicine, University of Otago Christchurch, New Zealand; 4 Professor, Department of Pathology, University of Otago Christchurch, New Zealand; 5 Economic and Social Research Council Future Research Leader Fellow, Division of Psychological Medicine and Clinical Neurosciences, Cardiff University and School of Psychology, University of Sussex, UK; 6 Research Associate, Division of Psychological Medicine and Clinical Neurosciences, Cardiff University, UK; 7 Division of Psychological Medicine and Clinical Neurosciences, Cardiff University, UK; 8 Research Associate, Population Health Sciences, Bristol Medical School and Medical Research Council Integrative Epidemiology Unit, University of Bristol, UK; 9 Professor, Institute of Health and Wellbeing, University of Glasgow, UK; 10 Professor, Division of Psychological Medicine and Clinical Neurosciences, Cardiff University and Population Health Sciences, Bristol Medical School, University of Bristol, UK; 11 Professor, Division of Psychological Medicine and Clinical Neurosciences, Cardiff University, UK; 12 Professor, Division of Psychological Medicine and Clinical Neurosciences, Cardiff University and School of Psychology, University of Sussex and School of Psychology, Trinity College, UK

**Keywords:** Schizophrenia, anxiety, polygenic risk score, CHDS, ALSPAC

## Abstract

**Background:**

Studies involving clinically recruited samples show that genetic liability to schizophrenia overlaps with that for several psychiatric disorders including bipolar disorder, major depression and, in a population study, anxiety disorder and negative symptoms in adolescence.

**Aims:**

We examined whether, at a population level, association between schizophrenia liability and anxiety disorders continues into adulthood, for specific anxiety disorders and as a group. We explored in an epidemiologically based cohort the nature of adult psychopathology sharing liability to schizophrenia.

**Method:**

Schizophrenia polygenic risk scores (PRSs) were calculated for 590 European-descent individuals from the Christchurch Health and Development Study. Logistic regression was used to examine associations between schizophrenia PRS and four anxiety disorders (social phobia, specific phobia, panic disorder and generalised anxiety disorder), schizophrenia/schizophreniform disorder, manic/hypomanic episode, alcohol dependence, major depression, and – using linear regression – total number of anxiety disorders. A novel population-level association with hypomania was tested in a UK birth cohort (Avon Longitudinal Study of Parents and Children).

**Results:**

Schizophrenia PRS was associated with total number of anxiety disorders and with generalised anxiety disorder and panic disorder. We show a novel population-level association between schizophrenia PRS and manic/hypomanic episode.

**Conclusions:**

The relationship between schizophrenia liability and anxiety disorders is not restricted to psychopathology in adolescence but is present in adulthood and specifically linked to generalised anxiety disorder and panic disorder. We suggest that the association between schizophrenia liability and hypomanic/manic episodes found in clinical samples may not be due to bias.

**Declarations of interest:**

None.

Schizophrenia is a debilitating, heritable disorder affecting between 0.5–1% of the population worldwide.^1^ Genetic factors contribute between 65–80% of the variance in risk and, of this, about a third to a half of the liability is distributed among many common risk alleles of small effect.^2^^–^^4^ At the individual level, the risk attributable to these alleles can be indexed by calculating polygenic risk scores (PRSs). Genetic studies of clinically ascertained cohorts have shown that schizophrenia PRS correlates with schizophrenia-affected status^3^^,^^5^ and also with other psychiatrically relevant traits, including bipolar disorder,^3^ major depression^6^ and poorer cognitive function.^7^^,^^8^ Studies demonstrating overlap between schizophrenia liability and other psychiatric disorders have generally been undertaken in samples that are not typical of disorders at the population level. For example, they are often enriched for severity, chronicity and attendance at specialist clinics. It is therefore important to determine whether findings from atypical samples generalise to samples ascertained to be representative of their source population. We employ two such samples in our study (New Zealand and UK). Previous studies employing the Avon Longitudinal Study of Parents and Children (ALSPAC),^9^^,^^10^ a birth cohort study based on children born in South West England (Avon; 1991–1992), demonstrate that schizophrenia PRSs were not associated with positive schizophrenia or depressive symptoms in adolescence (12–18 years), but was associated with negative symptoms and anxiety disorders.^11^ Hence, negative symptoms and anxiety may be more relevant to the transition to schizophrenia during adolescence than positive symptoms. Schizophrenia is frequently preceded by, and can be comorbid with, anxiety disorders;^12^ one reason for this is shared genetic aetiology. However, the ALSPAC study does not yet extend into adulthood and therefore it is unclear whether the pleiotropic effects of schizophrenia risk alleles on anxiety in the general population are transient, or whether they continue into adulthood. To clarify this, we used the Christchurch Health and Development Study (CHDS). The CHDS includes measures of mental health at multiple time points in adulthood;^13^^–^^15^ as these include multiple anxiety disorders (specific phobia, social phobia, panic disorders and generalised anxiety disorder [GAD]), we also investigated whether schizophrenia liability is increased across anxiety disorders or whether it shows any specificity to a particular diagnosis. We used the CHDS and ALSPAC data sets to investigate the population-level relationship between schizophrenia PRS and other forms of psychopathology: schizophrenia/schizophreniform disorder, manic/hypomanic episode, depression and alcohol dependence.

## Methods

### Sample

We employ two samples in our study. Our primary sample is the CHDS, which is an ongoing birth cohort of 1265 children born in the Christchurch (New Zealand) urban region in mid-1977. Participants have been studied on a total of 23 occasions from birth to age 35 years.^13^^,^^14^ The present analysis is based on data collected during assessments on the cohort at ages 21, 25, 30 and 35 years. Sample retention rates have remained high throughout the course of the study and, at age 35, the study was still able to assess 79% of the surviving cohort. Participant consent was obtained for all forms of data collection and all phases of the study have been subject to ethical approval. Ethical approvals relevant to the current research are from the Southern Health and Disability Ethics Committee (ref CTB/04/11/234/AM09, 16/STH/188/AM01).

We also employed the ALSPAC (www.bris.ac.uk/alspac/), which is a birth cohort study based on children born in South West England (Avon) between 1 April 1991 and 31 December 1992.^9^ Participants were not randomly selected, but the demographic characteristics of ALSPAC have been shown to be representative of the UK as a whole.^9^ The resource comprises a wide range of phenotypic and environmental measures in addition to biological samples, genetic (DNA on 11 343 children, genome-wide data on 8365 children and complete genome sequencing on 2000 children) and epigenetic (methylation sampling on 1000 children) information, and linkage to health and administrative records. The study website contains details of all the data, searchable through the data dictionary (www.bris.ac.uk/alspac/researchers/data-access/data-dictionary/). This study received ethical approval from the ALSPAC Law and Ethics Committee and Local Research Ethics Committees (http://www.bristol.ac.uk/alspac/researchers/research-ethics/).

### DNA extraction and genotyping

In the CHDS sample, individuals between the ages of 28–30 years provided a DNA sample for genetic analysis. In 91.4% of cases, DNA was extracted from whole blood; for the remaining participants (8.6%), saliva was collected using Oragene Collection Kits (DNA Genotek, Ottawa, Canada) and DNA was extracted according to the supplier's instructions. DNA extractions were completed at the Gene Structure and Function Laboratory, based at the University of Otago, Christchurch, New Zealand.^16^ Available samples were genotyped using Illumina Human660W-Quad v1 DNA Analysis BeadChips at the Mayo Clinic.^15^

Full details of sample and variant quality control are given in Supplementary Text and Supplementary Table 1 available at https://doi.org/10.1192/bjp.2018.227. Genotypes were imputed using the 1000 Genomes Phase 3 data set as a reference panel (see Supplementary Text). Principal component analysis was performed to produce population principal components for use as covariates in further analyses. The data set from which the risk alleles are derived are overwhelmingly of European ancestry so to maximise power and avoid confounding,^17^ analyses were limited to individuals of European ancestry as defined by genomic similarity to the European reference samples represented in the 1000 Genomes data set.^18^

### PRS construction

The PRS is a summary score of the number of risk alleles carried by an individual, weighted according to their effect sizes, where risk allele and effect size are determined from an independent genome-wide association study (GWAS) of the disorder (the ‘discovery set’).^3^ PRSs can predict schizophrenia status in case–control studies and capture sufficient risk to examine genetic relationships between different diagnostic categories.^5^

PRSs were derived using the method employed by the International Schizophrenia Consortium.^3^ Risk alleles and odds ratios were taken from the discovery data set made available by the Schizophrenia Working Group of the Psychiatric Genomics Consortium (PGC) which contained 35 476 cases of the disorder and 46 839 controls.^5^ To allow for the effects of linkage disequilibrium, variants were restricted to autosomal single-nucleotide polymorphisms (SNPs) in relative linkage equilibrium (using --clump in PLINK version 1.07 on a UNIX system^19^ with the PGC2 schizophrenia meta-analysis *P*-values, with a maximum *r*^2^ of 0.25 and a window size of 500 kb), leaving 87 713 SNPs. PRSs were calculated for each CHDS participant as the mean number of risk alleles surpassing a particular association *P*-value threshold (*P*_T_) in the PGC study, each weighted by its log-odds ratio, using the program PLINK.^19^ Primary analyses are based on scores constructed using a significance threshold (*P*_T_) of 0.05 in the discovery GWAS as this is the modal threshold that captures the maximal variance to the schizophrenia phenotype in each of the subsamples of that meta-analysis.^5^ The risk scores were divided into quartiles for further analysis. For phenotypes that showed significant relationships with schizophrenia PRSs, secondary analyses were performed across a range of *P*_T_ values (*P*_T_ = 1, 0.5, 0.3, 0.2, 0.1, 0.01, 0.001, 1 × 10^−4^, 1 × 10^−6^, 5 × 10^−8^) to ensure the pattern of results was not particularly sensitive to the chosen threshold. Secondary analyses were also performed on these phenotypes by using continuous PRS values to ensure that our results were not sensitive to our use of PRS quartiles as a primary measure.

### Mental health outcomes (18–35 years)

At ages 21, 25, 30 and 35 years, participants were administered a comprehensive mental health interview that assessed aspects of the individual's mental health and psychosocial adjustment over the period since the previous assessment. As part of this interview, the symptoms obtained were used to assign DSM-IV (1994)^20^ mood disorders (major depression, manic/hypomanic episode); anxiety disorders (GAD, panic disorders, agoraphobia, social phobia, specific phobia) and alcohol dependence. Questioning was based on the relevant sections of the Composite International Diagnostic Interview (CIDI),^21^ which is a structured interview for mental/psychiatric disorders. The CIDI has been used extensively in community and epidemiological surveys of disorder.^20^ From age 30 years, interviews were extended to include symptoms required to make DSM-IV diagnoses of schizophrenia/schizophreniform disorder. Interviews were administered by trained lay interviewers, as per the design of the CIDI. In the majority of cases (approximately 80%), interviews were conducted face to face or, if this was not possible (e.g. the participant was resident overseas), via telephone or Skype. Using these data, participants were first classified as to whether they met criteria for each of the above diagnoses in each of the interview periods from age 18–21, 21–25, 25–30 and 30–35 years. This information was then combined over interview periods to classify participants as to whether they had ever met criteria for the DSM-IV diagnoses over the whole period of age 18–35 years. The diagnoses included in the present analysis and associated adult lifetime (18–35 years) prevalence estimates were as follows: major depression (47.6%), manic or hypomanic episode (7.6%), GAD (9.1%), panic disorder with or without agoraphobia (14.2%), social phobia (15.4%), specific phobia (20.7%), alcohol dependence (12.9%) and schizophrenia/schizophreniform disorder (2.0%). The total number of anxiety disorders (defined as specific phobia, social phobia, panic disorder and GAD) was also calculated and used as a phenotypic variable. In this longitudinal study of lifetime adult disorder (up to age 30), consistent with aetiological pleiotropy, there was substantial comorbidity between domains of mood disorder (e.g., major depressive disorder, manic/hypomanic episode), anxiety, alcohol dependence and schizophrenia (odds ratios up to 30 depending on disorder; see Supplementary Table 2).

### Sample size and statistical analysis

The present analysis was based on a sample of 590 participants (286 male and 304 female) from CHDS who were observed on mental health outcomes from age 18 to 35 years, who were successfully genotyped on the Illumina chip and therefore for whom schizophrenia PRSs could be constructed. As above, analyses were limited to participants who were of European ancestry. Comparison of those included in the analysis with those not included showed no statistically significant differences in the observed rates of disorder (see Supplementary Text and Supplementary Table 3).

We performed regression of the phenotype variables on quartiles of schizophrenia PRSs (logistic regression for dichotomous outcomes, linear regression for the number of anxiety disorders). Gender and the first two population principal components were included in the regression as covariates. Analyses were conducted using the glm() function in R.^22^ As phenotype measures were correlated, Bonferroni multiple testing correction would be overly conservative and so we used false discovery rate (FDR) multiple testing correction (in R) instead.^23^ Where significant associations were found between schizophrenia PRS and anxiety disorders, conditional analysis was conducted to determine whether these associations were independent of each other.

To examine the effect of comorbidity among anxiety disorders, we also performed a secondary association analysis comparing individuals with any one anxiety disorder with those with no anxiety disorders, and another comparing individuals with two or more anxiety disorders with those with no anxiety disorders (see Supplementary Table 2).

### Replication sample and analysis

To replicate our novel finding from the CHDS study, we tested the relationship between schizophrenia PRS and mania/hypomania episode in the ALSPAC sample. The initial cohort contained 15 445 participants with extensive baseline information from the first trimester of pregnancy onwards. Data were collected regularly at defined time intervals and is ongoing.

Hypomanic features in ALSPAC was assessed using the Hypomania Checklist (HCL-32) at age 22–23 years. The HCL-32 is a self-rating questionnaire designed to capture a lifetime history of hypomanic symptoms.^24^^–^^27^ It has been used extensively in clinical and non-clinical settings and is validated as a screening tool for bipolar disorder type II. Following a Rasch analysis for unidimensionality of the HCL-32, four items were identified as redundant and could be excluded.^28^ Our primary analysis in ALSPAC was based on the preferred definition of hypomanic episode in ALSPAC, which is defined as meeting a threshold score on the HCL-32 of 14 or more (out of 28), having symptoms for at least 2 days and a response of either a negative or both negative and positive impact of periods of elevated mood on family, social, or work life or on leisure. However, we tested a more stringent definition requiring a 4 day duration, as in CHDS.

Genetic data from 9912 participants were obtained using a genome-wide SNP genotyping platform (HumanHap550-Quad; Illumina). Following quality control, imputation and restriction to 1 young person per family, genetic data were available on 8230 individuals, of whom 2655 individuals were of European ancestry and had HCL-32 data. Based on the 2-day duration criterion, the number of individuals classed as having a hypomanic episode (*n* = 239) was comparable to that in CHDS (~7%), but this fell to 50 (1.88%) when the 4-day criterion was applied.

The schizophrenia PRS was constructed in the same way as for the CHDS sample (see above), using the PGC2 schizophrenia study as the discovery data set^5^ and a *P*_T_ threshold of 0.05. The relationship between schizophrenia PRS and hypomanic episode was examined using logistic regression in STATA (v14.1 SE, Macintosh platform), including gender as a covariate. In both the CHDS and ALSPAC samples, schizophrenia PRSs were calculated blind to phenotype classification.

## Results

**Fig. 1:**
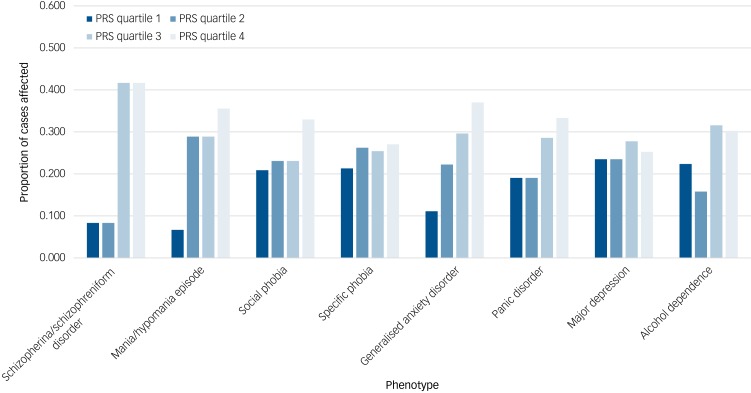
Proportion of cases affected at schizophrenia polygenic risk score quartiles 1–4 for each phenotype in the Christchurch Health and Development Study (CHDS). PRS, polygenic risk score.

**Fig. 2:**
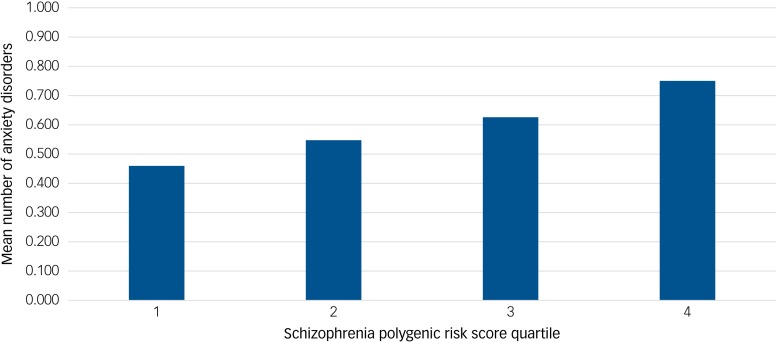
Mean number of anxiety disorders per sample at schizophrenia polygenic risk score (SZ PRS) quartile 1–4 in the Christchurch Health and Development Study (CHDS) sample.

Results for the CHDS sample are given in Table 1. The proportion of cases affected by each dichotomous phenotype within each quartile of schizophrenia PRSs are given in Fig. 1. Despite the very small number of individuals (*n* = 12) meeting criteria for schizophrenia/schizophreniform disorder, higher schizophrenia PRS was associated with this phenotype, although evidence for this was weaker after correction for multiple testing (FDR-corrected *P* = 0.052). Across anxiety disorders, schizophrenia PRS was associated both with GAD (FDR-corrected *P* = 0.018) and panic disorder (FDR-corrected *P* = 0.034) but not with specific or social phobias. When the association between schizophrenia PRS and GAD was conditioned on panic disorder, the result was no longer significant (FDR-corrected *P* = 0.086). The same was true when the association between schizophrenia PRS and panic disorder was conditioned on GAD (FDR-corrected *P* = 0.22). Among the other phenotypes, higher schizophrenia PRS was associated with manic/hypomanic episode (FDR-corrected *P* = 0.021), but not with depression or alcohol dependence. Schizophrenia PRS was also associated with number of anxiety disorders (FDR-corrected *P* = 0.018; Fig. 2). When we performed separate association analyses on cases with a single anxiety disorder and those with two or more, the latter were much more significantly associated with schizophrenia PRS (Supplementary Table 4).

**Table 1:** Association between schizophrenia polygenic risk score quartile and phenotype in Christchurch Health and Development Study sample

Phenotype	Affected sample count	Unaffected sample count	Odds ratio	95% CI	*P*-value	*P*-value (FDR corrected)	Nagelkerke's *R*^2^
Schizophrenia/schizophreniform disorder	12	578	1.906	1.092, 3.688	0.029	0.052	0.026
Manic/hypomanic episode	45	545	1.499	1.129, 2.024	0.007	0.021	0.025
Social phobia	91	499	1.226	0.997, 1.514	0.059	0.089	0.052
Specific phobia	122	468	1.109	0.924, 1.334	0.305	0.321	0.053
Generalised anxiety disorder	54	536	1.502	1.153, 1.984	0.004	0.018	0.048
Panic disorder	84	506	1.280	1.034, 1.592	0.015	0.034	0.048
Major depression	281	309	1.067	0.921, 1.237	0.321	0.321	0.051
Alcohol dependency	76	514	1.223	0.983, 1.529	0.092	0.118	0.029
Phenotype	Affected sample count^a^	Unaffected sample count	Interquartile disorder count increase^b^	95% confidence intervals	*P*-value	*P*-value (FDR corrected)	Nagelkerke's *R*^2^
Total number of anxiety disorders	208	382	0.104	0.033, 0.174	0.004	0.018	0.084

Linear regression used for ‘Total number of anxiety disorders’, logistic regression used for all other phenotypes.

a. This indicates number of samples with one or more of the anxiety disorders measured here (generalised anxiety disorder, panic disorder, social phobia and specific phobia).

b. This is defined as the expected increase in number of anxiety disorders for a unit increase in schizophrenia polygenic risk score quartile.

*P*-value threshold of 0.05.

All phenotypes showing significant associations with schizophrenia PRSs in our primary analysis (*P*_T_ = 0.05) also showed evidence for association across multiple thresholds, which shows our results were not highly sensitive to our choice of this threshold (see Supplementary Table 5). In some instances, the associations were much more significant at an alternative threshold. For example, for total number of anxiety disorders the strongest evidence for association (*P* = 8.1 × 10^−6^) was observed at *P*_T_ = 0.01.

Similarly, when we used continuous PRSs instead of PRS quartiles as a secondary analysis, all phenotypes showing significant associations with schizophrenia PRS remained significantly associated, which shows our results are not sensitive to our decision to use PRS quartiles (see Supplementary Table 6).

Finally, we confirmed our observation of association between manic/hypomanic episode and schizophrenia PRS in ALSPAC (*P* of 0.002 for symptom duration criterion of 2 days, *P* of 0.005 for symptom duration criterion of 4 days).

## Discussion

This study investigated the relationship between schizophrenia genetic liability and psychiatric diagnoses in early adulthood in a New Zealand population cohort. We found associations between schizophrenia PRS and manic/hypomanic episodes and anxiety disorders, but other phenotypes were either not significantly associated, or, in the case of schizophrenia/schizophreniform disorder, did not survive correction for multiple testing.

### Schizophrenia PRS and schizophrenia

An initial association between schizophrenia PRS and schizophrenia/schizophreniform disorder did not remain significant following correction for multiple testing (Table 1). These inconclusive results are consistent with previous population studies in adolescents which found no evidence of association between schizophrenia PRS and psychotic experiences. The symptoms included in assigning schizophrenia/schizophreniform diagnoses here included both negative and positive symptoms, which may explain the weak evidence for association, as a previous study (based on ALSPAC) found an association between PRS and negative symptoms but not positive symptoms.^11^

The low frequency of schizophrenia/schizophreniform diagnoses in our sample (2.0%) compared with GAD (9.2%) and manic/hypomanic episode (7.6%) is a likely reason for the weak evidence for association observed here. Consistent with this and our expectations, it is notable that the point estimate for the effect size for association (odds ratio of 1.906) was stronger for schizophrenia/schizophreniform than for any other disorder. In addition to a genuine relative rarity of schizophrenia/schizophreniform in the population compared with other diagnoses, we also note the symptoms required to assign that diagnosis were only obtained at two time points in the CHDS study (30–35 years), whereas those required to assign other diagnoses were measured across four time points. Thus, it is possible we have missed a higher fraction of true schizophrenia/schizophreniform diagnoses than may be the case for other diagnoses.

### Schizophrenia PRS and anxiety disorders

We found strong evidence of association between schizophrenia PRS and GAD, and schizophrenia PRS and panic disorder (Table 1). A higher schizophrenia PRS was also strongly associated with total number of anxiety disorders.

Anxiety disorders in adolescence were associated with schizophrenia PRS in the ALSPAC data set, suggesting that anxiety may be a prodromal feature in schizophrenia.^11^ Similarly, the associations between schizophrenia PRS, GAD and panic disorder show that the relationship between schizophrenia genetic risk and anxiety persists into adulthood. Whether this relationship reflects direct causal overlap between the disorders or pleiotropic effects of schizophrenia-associated genes on anxiety disorder is unclear. As a next step, it may be interesting to examine whether this genetic overlap is driven by genes belonging to specific biological pathways, particularly those with some evidence for involvement in schizophrenia.

The conditional analysis suggested that the GAD result was not conditionally independent of panic disorder and vice versa. Alongside the relationship between schizophrenia PRS and total number of anxiety disorders, this suggests that as anxiety disorder comorbidity increases, so does the relevance of schizophrenia genetic risk. This is further emphasised by the secondary analysis showing that almost all of the association signal for schizophrenia PRS comes from cases with two or more anxiety disorders (Supplementary Table 4).

### Schizophrenia PRS and other psychopathological variables

We also found a positive association between schizophrenia PRS and manic/hypomanic episode in the CHDS sample. This positive association was replicated in the ALSPAC population study, suggesting that it is unlikely to be due to chance. The links between schizophrenia and bipolar disorder are well documented at both the phenotypic and genetic levels, with many individuals with schizophrenia showing manic symptoms and many individuals with bipolar disorder displaying psychotic symptoms. However, no previous population study has shown evidence of a relationship between schizophrenia genetic risk and manic/hypomanic episode. Thus this finding is a novel demonstration of genetic overlap between the two disorders across the adult population beyond clinical populations.

Although anxiety and manic/hypomanic episode may be affected by schizophrenia genetic risk, it is unclear the extent to which they show a specific relationship with schizophrenia. Anxiety is a common symptom in a wide variety of psychiatric disorders, whereas mania and hypomania are the key features of bipolar disorder. The extent to which these symptoms and schizophrenia genetic risk index risk for a range of psychiatric disorders, including schizophrenia, is not yet fully understood. It is also unclear how these phenotypes and schizophrenia PRS affect specific symptoms or cognitive deficits. However, work with PRSs and linkage disequilibrium (LD) score techniques shows that there is considerable genetic overlap between different psychiatric disorders.^29^

### Strengths and limitations

This study has limitations besides those discussed above. The CHDS sample size is not as large as the ALSPAC or other relevant studies, so the sample may be insufficiently powered to detect true associations of small effect size. Generalisability of findings should also be considered relative to the sample attributes of both the New Zealand and UK cohorts included in the present study. The CHDS analysis presented here is based on European-descent samples from New Zealand, and so results may not generalise to other data sets from countries with different healthcare systems and cultural features. However, the presence of similar results in the ALSPAC cohort from the UK mitigates this issue considerably. As with all longitudinal cohort studies, there is also the possibility of different levels of attrition between groups of samples. A greater chance of non-response or missing data due to psychiatric symptoms for samples with high schizophrenia PRS would reduce power to find an association between the PRS and those symptoms.

One of the strengths of the study is that the PRS discovery set, the PGC2 meta-analysis (*n* = 82 315), is the largest publicly available, which should minimise measurement error in the scores and hence improve the power of studies using these scores for analyses in target samples.^5^ The CHDS sample is also a relatively richly phenotyped sample, which enables us to examine effects on specific anxiety disorders rather than only anxiety disorders as a whole.

### Impact of this study and future directions

This study shows a positive association between schizophrenia PRS and anxiety in adulthood where previous associations covered adolescence, suggesting that schizophrenia genetic risk can have an impact on anxiety aetiology in the absence of a clinical diagnosis of schizophrenia. It also shows greater specificity than previous work, which only found association between the presence of any anxiety disorder and schizophrenia PRS. Although all anxiety disorders considered here make some contribution to the association, the strongest drivers of this effect are GAD and panic disorder. We also showed association between schizophrenia PRS and manic/hypomanic episodes in a population cohort, emphasising the overlap between these two disorders. Further work in larger population cohorts with measures of adult psychiatric dysfunction would be useful to replicate these results.

## Supplementary material

For supplementary material accompanying this paper visit https://doi.org/10.1192/bjp.2018.227. click here to view supplementary material
