# Supplementary material for: Associations between schizophrenia genetic risk, anxiety disorders and manic/hypomanic episode in a longitudinal population cohort study
Source: Br J Psychiatry. 2019 Feb;214(2):96–102. doi: 10.1192/bjp.2018.227 (PMC6429243; doi:10.1192/bjp.2018.227)
Supplement: Supplementary file 1 [file S0007125018002271sup001.docx]

**Supplementary tables**

| **Sample exclusion criteria** | **Exclusion thresholds** |
| --- | --- |
| Sample call rate | Call rate < 99% |
| Heterozygosity | F < -0.01, > 0.015 |
| Relative check | PI-HAT > 0.2 |
| PCA | PC1 < 0.008, PC2 < 0.0135 |
|  |  |
|  |  |
| **SNP exclusion criteria** | **Exclusion thresholds** |
| SNP call rate | Call rate < 99% |
| Hardy-Weinberg Equilibrium | HWE p < 1e-6 |

Supplementary Table 1. Sample and variant quality control and European ancestry exclusion criteria in CHDS sample.

| Outcome  Predictor | Manic/ hypomanic episode (95% CI) | Social phobia  OR (95% CI) | Depression OR (95% CI) | GAD OR (95% CI) | Panic disorder  OR (95% CI) | Specific phobia OR (95% CI) | Alcohol dependency OR (95% CI) | SZ/SZP disorder OR (95% CI) |
| --- | --- | --- | --- | --- | --- | --- | --- | --- |
| Manic/hypomanic episode | -- | 4.80 (2.5,9.09) | 5.71 (2.74,13.4) | 8.34 (4.14,16.53) | 3.06 (1.51,5.95) | 3.85 (2.05,7.19) | 2.39 (1.11,4.81) | 19.89 (6.07,70.06) |
| Social phobia |  | -- | 6.14 (3.6,11.03) | 9.73 (5.37,17.87) | 10.74 (6.39,18.23) | 5.36 (3.33,8.66) | 2.23 (1.24,3.89) | 30.68 (7.91,201.97) |
| Depression |  |  | -- | 10.40 (4.72,27.51) | 6.99 (3.95,13.24) | 2.53 (1.68,3.86) | 3.60 (2.13,6.3) | 3.37 (1,15.33) |
| GAD |  |  |  | -- | 9.23 (5.07,16.94) | 3.28 (1.82,5.84) | 2.38 (1.17,4.58) | 11.04 (3.34,36.59) |
| Panic disorder |  |  |  |  | -- | 4.15 (2.54,6.77) | 2.30 (1.26,4.06) | 13.21 (4.06,50.48) |
| Specific phobia |  |  |  |  |  | -- | 2.11 (1.23,3.54) | 5.64 (1.77,19.35) |
| Alcohol dependency |  |  |  |  |  |  | -- | 0.00 |
| SZ/SZP disorder |  |  |  |  |  |  |  | -- |

Supplementary Table 2. Logistic regression examining associations between phenotype variables within CHDS sample.

| **Phenotype** | **Odds ratio** | **P-value (uncorrected)** |
| --- | --- | --- |
| Schizophrenia/schizophreniform disorder | 1.86 | 0.0410 |
| Mania/hypomania episode | 1.50 | 0.0060 |
| Social phobia | 1.23 | 0.0557 |
| Specific phobia | 1.11 | 0.2681 |
| Generalised anxiety disorder | 1.50 | 0.0034 |
| Panic disorder | 1.28 | 0.0245 |
| Major depression | 1.06 | 0.4033 |
| Alcohol dependency | 1.22 | 0.0723 |
| **Phenotype** | **Interquartile disorder count increase** | **P-value (uncorrected)** |
| Total number of anxiety disorders | 0.10 | 0.0043 |

Supplementary Table 3. Association between SZ PRS quartile and phenotypes using sample weights to correct for selection bias in CHDS sample. Linear regression used for ‘Total number of anxiety disorders’, logistic regression used for all other phenotypes. Interquartile disorder count increase is defined as the expected increase in number of anxiety disorders for a unit increase in SZ PRS quartile.

| **Phenotype** | **P value threshold** | **Odds ratio** | **Association p-value** |
| --- | --- | --- | --- |
| One anxiety disorder | 0.05 | 1.003 | 0.973 |
| Two or more anxiety disorders | 0.05 | 1.367 | 0.005 |

Supplementary Table 4. Association between SZ PRS quartile and presence of either a single anxiety disorder or multiple anxiety disorders (using individuals with no anxiety disorders as controls) in CHDS sample. Logistic regression used for analysis.

| **Phenotype** | **P value threshold** | **Odds ratio** | **Association p-value** |
| --- | --- | --- | --- |
| GAD | 1 | 1.483 | 0.004 |
| GAD | 0.5 | 1.431 | 0.008 |
| GAD | 0.3 | 1.407 | 0.011 |
| GAD | 0.2 | 1.414 | 0.010 |
| GAD | 0.1 | 1.595 | 0.001 |
| GAD | 0.05 | 1.431 | 0.004 |
| GAD | 0.01 | 1.862 | 2.76E-05 |
| GAD | 1.00E-04 | 1.377 | 0.017 |
| GAD | 1.00E-06 | 1.409 | 0.012 |
| GAD | 5.00E-08 | 1.383 | 0.016 |
| Mania/hypomania episode | 1 | 1.734 | 3.60E-04 |
| Mania/hypomania episode | 0.5 | 1.657 | 0.001 |
| Mania/hypomania episode | 0.3 | 1.653 | 0.001 |
| Mania/hypomania episode | 0.2 | 1.586 | 0.002 |
| Mania/hypomania episode | 0.1 | 1.481 | 0.008 |
| Mania/hypomania episode | 0.05 | 1.657 | 0.007 |
| Mania/hypomania episode | 0.01 | 1.705 | 0.001 |
| Mania/hypomania episode | 1.00E-04 | 1.510 | 0.005 |
| Mania/hypomania episode | 1.00E-06 | 1.240 | 0.133 |
| Mania/hypomania episode | 5.00E-08 | 1.231 | 0.144 |
| Panic disorder | 1 | 1.280 | 0.025 |
| Panic disorder | 0.5 | 1.278 | 0.026 |
| Panic disorder | 0.3 | 1.303 | 0.017 |
| Panic disorder | 0.2 | 1.302 | 0.016 |
| Panic disorder | 0.1 | 1.298 | 0.018 |
| Panic disorder | 0.05 | 1.278 | 0.015 |
| Panic disorder | 0.01 | 1.417 | 0.002 |
| Panic disorder | 1.00E-04 | 1.282 | 0.023 |
| Panic disorder | 1.00E-06 | 1.318 | 0.012 |
| Panic disorder | 5.00E-08 | 1.205 | 0.085 |
| **Phenotype** | **P value threshold** | **Interquartile disorder count increase** | **Association p-value** |
| Total number of anxiety disorders | 1 | 0.105 | 0.002 |
| Total number of anxiety disorders | 0.5 | 0.095 | 0.005 |
| Total number of anxiety disorders | 0.3 | 0.109 | 0.004 |
| Total number of anxiety disorders | 0.2 | 0.118 | 0.008 |
| Total number of anxiety disorders | 0.1 | 0.108 | 0.001 |
| Total number of anxiety disorders | 0.05 | 0.104 | 0.004 |
| Total number of anxiety disorders | 0.01 | 0.097 | 8.11E-06 |
| Total number of anxiety disorders | 1.00E-04 | 0.100 | 0.005 |
| Total number of anxiety disorders | 1.00E-06 | 0.103 | 0.004 |
| Total number of anxiety disorders | 5.00E-08 | 0.076 | 0.040 |

Supplementary Table 5. Association between SZ PRS quartile and GAD, manic/hypomanic symptoms, panic disorder and total number of anxiety disorders in CHDS sample. Range of P_T_ values used: P_T_=1, 0.5, 0.3, 0.2, 0.1, 0.05, 0.01, 1x10^-4^, 1x10^-6^, 5x10^-8^ in CHDS sample. Linear regression used for ‘Total number of anxiety disorders’, logistic regression used for all other phenotypes. Interquartile disorder count increase is defined as the expected increase in number of anxiety disorders for a unit increase in SZ PRS quartile.

| **Phenotype** | **P value threshold** | **Odds ratio** | **Association p-value** |
| --- | --- | --- | --- |
| GAD | 1 | 1.585 | 0.003 |
| GAD | 0.5 | 1.547 | 0.005 |
| GAD | 0.3 | 1.562 | 0.004 |
| GAD | 0.2 | 1.557 | 0.004 |
| GAD | 0.1 | 1.622 | 0.002 |
| GAD | 0.05 | 1.578 | 0.003 |
| GAD | 0.01 | 1.835 | 1.29E-04 |
| GAD | 1.00E-04 | 1.391 | 0.027 |
| GAD | 1.00E-06 | 1.551 | 0.007 |
| GAD | 5.00E-08 | 1.469 | 0.019 |
| Mania/hypomania episode | 1 | 1.836 | 3.90E-04 |
| Mania/hypomania episode | 0.5 | 1.785 | 0.001 |
| Mania/hypomania episode | 0.3 | 1.761 | 0.001 |
| Mania/hypomania episode | 0.2 | 1.683 | 0.002 |
| Mania/hypomania episode | 0.1 | 1.629 | 0.004 |
| Mania/hypomania episode | 0.05 | 1.615 | 0.004 |
| Mania/hypomania episode | 0.01 | 1.701 | 0.001 |
| Mania/hypomania episode | 1.00E-04 | 1.449 | 0.021 |
| Mania/hypomania episode | 1.00E-06 | 1.433 | 0.037 |
| Mania/hypomania episode | 5.00E-08 | 1.421 | 0.044 |
| Panic disorder | 1 | 1.304 | 0.035 |
| Panic disorder | 0.5 | 1.302 | 0.037 |
| Panic disorder | 0.3 | 1.282 | 0.049 |
| Panic disorder | 0.2 | 1.318 | 0.029 |
| Panic disorder | 0.1 | 1.312 | 0.032 |
| Panic disorder | 0.05 | 1.320 | 0.028 |
| Panic disorder | 0.01 | 1.484 | 0.002 |
| Panic disorder | 1.00E-04 | 1.396 | 0.007 |
| Panic disorder | 1.00E-06 | 1.390 | 0.012 |
| Panic disorder | 5.00E-08 | 1.330 | 0.028 |
| **Phenotype** | **P value threshold** | **Effect size (beta)** | **Association p-value** |
| Total number of anxiety disorders | 1 | 0.120 | 0.003 |
| Total number of anxiety disorders | 0.5 | 0.117 | 0.004 |
| Total number of anxiety disorders | 0.3 | 0.110 | 0.006 |
| Total number of anxiety disorders | 0.2 | 0.118 | 0.004 |
| Total number of anxiety disorders | 0.1 | 0.125 | 0.002 |
| Total number of anxiety disorders | 0.05 | 0.118 | 0.004 |
| Total number of anxiety disorders | 0.01 | 0.161 | 5.98E-05 |
| Total number of anxiety disorders | 1.00E-04 | 0.118 | 0.003 |
| Total number of anxiety disorders | 1.00E-06 | 0.123 | 0.002 |
| Total number of anxiety disorders | 5.00E-08 | 0.093 | 0.019 |

Supplementary Table 6. Association between continuous SZ PRS values and GAD, manic/hypomanic symptoms, panic disorder and total number of anxiety disorders. Range of P_T_ values used: P_T_=1, 0.5, 0.3, 0.2, 0.1, 0.05, 0.01, 1x10^-4^, 1x10^-6^, 5x10^-8^ in CHDS sample. Linear regression used for ‘Total number of anxiety disorders’, logistic regression used for all other phenotypes.

**Supplementary Text**

**Genotype quality control**

The samples of the CHDS dataset were filtered for call rate (>99% required for inclusion) and heterozygosity (F between -0.01 and 0.015 required for inclusion). The variants were filtered for call rate (>99% required) and Hardy-Weinberg equilibrium (HWE p>1e^-6^ required). Monomorphic variants and variants with MAF<1% were also excluded. Genotypic sex was determined using PLINK, and 2 samples where database sex did not match were excluded. IBD was used to remove relatives with PI-HAT > 0.2. See Supplementary Table 1 for filtering thresholds. The dataset was restricted to variants in relative linkage equilibrium with each other (using --indep-pairwise in PLINK, with a maximum r2 of 0.25 and a window size of 500kb), and principal component analysis was performed to produce population principal components for use as covariates in further analyses. Principal component analysis of this cohort alongside the Thousand Genomes reference panel was also performed to determine which samples were of European ancestry (Supplementary Table 1).

**Imputation method**

After quality control, imputation was carried out on autosomal chromosomes using the software packages SHAPEIT to perform pre-phasing haplotype estimation and IMPUTE2 to perform the imputation (chunk size of 2MB). The 1000 Genomes Phase 3 dataset was used as a reference panel (October 2014 release). Variants with an INFO of 0.7 or above were retained for further analysis. PRS calculation was based on the dosage data for these variants.

**Additional P_T_ thresholds**

As a secondary analysis, PRS were calculated across a range of P_T_ thresholds for variables that showed significant association at P_T_=0.05 after multiple testing correction (GAD, panic disorder, mania/hypomania episode and total number of anxiety disorders – Supplemental Table 2). The thresholds used were the same as the PGC2 study (P_T_=1, 0.5, 0.3, 0.2, 0.1, 0.05, 0.01, 1x10-4, 1x10-6, 5x10-8). The results were robust to choice of P_T_, with the same direction of effect as the primary analysis throughout and significant associations (p<0.05) in the majority of cases. P_T_=0.01 produces particularly significant results across these four variables.

**Testing for sample selection bias**

The analyses reported in the paper were based on a sample of 590 participants identified as being of sole European ancestry for whom SZ polygenic risk scores were calculated and who were observed on adult mental health outcomes. This sample represented 76% of the 780 participants who identified as sole European and who provided DNA to the study, and 68% of the total sample still available to the study at age 28-30. The sources of sample loss have been described in the paper. The rate of sample attrition raises issues concerning the potential for sample selection or attrition bias to have influenced the study findings. To examine this issue the analysis sample were compared with the remainder of the surviving cohort on a range of measures of sample characteristics. These comparisons showed the presence of statistically significant (P<0.05) tendencies for the analysis sample to underrepresent participants from more socioeconomically disadvantaged backgrounds (low parental education, low SES families). However, these factors were not associated with the rates of adult mental health outcomes; nor were they correlated with SZ polygenic risk scores within the analysis sample. Further, inclusion/exclusion from the analysis sample was similarly unrelated to mental health outcomes. This suggests that the processes of sample attrition were unlikely to have impugned the validity of the analysis. This was confirmed by reanalysis of the data with the analysis sample weighted to correct for possible selection bias (1). In all cases the findings from the weighted analysis were consistent with those from the unweighted analysis (see Supplementary Table 3).

1. Little RJA, Rubin, D.B. Statistical Analysis with Missing Data. Wiley, 2002.
